# Designer laying hen diets to improve egg fatty acid profile and maintain sensory quality

**DOI:** 10.1002/fsn3.47

**Published:** 2013-06-14

**Authors:** Erin M Goldberg, Donna Ryland, Robert A Gibson, Michel Aliani, James D House

**Affiliations:** 1 Department of Human Nutritional Sciences, University of ManitobaWinnipeg, Manitoba, R3T 2N2, Canada; 2 Department of Nutrition and Functional Food Science, University of AdelaideAdelaide, South Australia, 5000, Australia; 3 Department of Animal Science, University of ManitobaWinnipeg, Manitoba, R3T 2N2, Canada

**Keywords:** Descriptive analysis, docosahexaenoic acid, eggs, laying hens, omega-3, saturated fat, sensory

## Abstract

The fatty acid composition of eggs is highly reflective of the diet of the laying hen; therefore, nutritionally important fatty acids can be increased in eggs in order to benefit human health. To explore the factors affecting the hen's metabolism and deposition of fatty acids of interest, the current research was divided into two studies. In Study 1, the fatty acid profile of eggs from Bovan White hens fed either 8%, 14%, 20%, or 28% of the omega-6 fatty acid, linoleic acid (LA) (expressed as a percentage of total fatty acids), and an additional treatment of 14% LA containing double the amount of saturated fat (SFA) was determined. Omega-6 fatty acids and docosapentaenoic acid (DPA) in the yolk were significantly (*P* < 0.05) increased, and oleic acid (OA) and eicosapentaenoic acid (EPA) were significantly decreased with an increasing dietary LA content. In Study 2, the fatty acid and sensory profiles were determined in eggs from Shaver White hens fed either (1) 15% or 30% of the omega-3 fatty acid, alpha-linolenic acid (ALA) (of total fatty acids), and (2) low (0.5), medium (1), or high (2) ratios of SFA: LA+OA. Increasing this ratio resulted in marked increases in lauric acid, ALA, EPA, DPA, and docosahexaenoic acid (DHA), with decreases in LA and arachidonic acid. Increasing the dietary ALA content from 15% to 30% (of total fatty acids) did not overcome the DHA plateau observed in the yolk. No significant differences (*P* ≥ 0.05) in aroma or flavor between cooked eggs from the different dietary treatments were observed among trained panelists (*n* = 8). The results showed that increasing the ratio of SFA: LA+OA in layer diets has a more favorable effect on the yolk fatty acid profile compared to altering the LA content at the expense of OA, all while maintaining sensory quality.

## Introduction

Linoleic acid (LA) and alpha-linolenic acid (ALA) are essential fatty acids found abundantly in plant sources that must be obtained in the human diet. LA and ALA are the parent compounds of the omega-6 and omega-3 polyunsaturated fatty acid (PUFA) series, respectively. The health benefits of consuming omega-3 PUFAs are well established (Yashodhara et al. 2009). In particular, consumption of omega-3 long-chain PUFAs (LCPUFAs) such as eicosapentaenoic acid (EPA), docosapentaenoic acid (DPA), and docosahexaenoic acid (DHA), typically found in fish and algae, offer cardiometabolic, immunologic, and neurologic protection, as reviewed by Innis et al. (2013). The current recommendation to prevent deficiency symptoms is between 0.6% and 1.2% omega-3 PUFAs of total energy, with up to 10% of this range composed of EPA and DHA (Health Canada 2012c). Humans have the ability to convert ALA into omega-3 LCPUFAs through a series of desaturation and elongation enzymatic reactions. Specifically, delta-5 and delta-6 desaturase enzymes (EC 1.14.19.3) are necessary to complete the process. However, conversion efficiency of ALA decreases down the elongation–desaturase cascade; therefore, DHA synthesis from ALA (∼0.5%) is even more restricted than that of EPA (∼5%) (Goyens et al. 2006; Plourde and Cunning 2007; Brenna et al. 2009). Furthermore, higher conversion rates in women have been reported, possibly due to the activation of the peroxisomal pathway by estrogens (Burdge 2004; Giltay et al. 2004).

The omega-6:omega-3 PUFA ratio in the diet plays an important role in health and disease (Myers and Allen 2012). The mechanism behind this can be attributed to the prothrombotic, proaggregatory, and proinflammatory properties of the eicosanoid metabolic products of omega-6 PUFAs as compared to the opposing physiological functions of omega-3 PUFAs; therefore, reducing this ratio in the diet is favorable (Simopoulos 2002, 2008; de Lorgeril and Salen 2012). Additionally, it has been found that there are several dietary factors which affect the conversion of ALA into LCPUFAs, including the LA:ALA ratio. It is suggested that because LA and ALA compete for the same rate-limiting enzymes, delta-5 and -6 desaturases, decreasing the LA:ALA ratio is important to encourage greater EPA and DHA synthesis (Simopoulos 2010). However, others have suggested that the absolute amount of dietary ALA is more indicative of the degree of ALA conversion rather than the ratio (Goyens et al. 2006).

As consumption of omega-3 PUFAs in the Western diet is limited, particularly LCPUFAs, developing alternative ways to increase consumption is critical. The use of hen eggs to serve this function is attractive for several reasons. Most importantly, the hen liver is able to convert a substantial proportion of dietary ALA into LCPUFAs that can be deposited in the egg yolk (Gonzalez-Esquerra and Leeson 2001). Because of this, vegetarian ingredients can be used in the feed rather than unsustainable marine products which may also be contaminated with mercury (Health Canada 2012a). In harnessing the hen's natural ability to convert ALA into LCPUFAs, this functional food could serve a critical role in the health of individuals who do not consume seafood for various reasons. Additionally, it is well known that the incorporation of omega-3 PUFAs into eggs may result in the development of undesirable aroma and flavor attributes. Therefore, sensory evaluation is an essential component in determining any potential differences in these eggs.

The majority of studies in this area have focused on the resulting egg fatty acid composition through the inclusion of particular ingredients, such as fish, algae, or oilseeds, rather than controlling for dietary effects by manipulating the dietary fatty acid composition. Our previous work has demonstrated that a decreased LA:ALA ratio in the laying hen diet results in increased yolk LCPUFAs, eventually reaching a DHA plateau (Goldberg et al. 2012). Therefore, the objectives of this research were to incorporate oils and fats in varying amounts in order to maximize ALA conversion and deposition of fatty acids of interest into resultant eggs while assessing the potential for any sensory changes that may occur.

## Materials and Methods

This research was divided into two studies. The objectives of each study are listed below:

1. Study 1 – To determine the impact of LA in the layer diet on yolk fatty acids.
2. Study 2 – To determine the impact of saturated fat (SFA) in the layer diet on yolk fatty acids.

The main purpose of both studies was to determine the extent to which these dietary fats influence the conversion of ALA to DHA into egg yolk if competition between omega-3 and -6 fatty acids for access to delta-5 and -6 desaturase enzymes is removed. By maintaining a constant ALA level in our diets in both Studies 1 and 2, we were able to assess this more effectively. Additionally, it is important that sensory quality be maintained in order to remain palatable to consumers; therefore, it would be a desired outcome if the aroma and flavor of these eggs remain unaffected despite the expected fatty acid alteration.

### Bird housing and environment

Forty Bovan White hens at 49 weeks of age and 48 Shaver White laying hens at 37 weeks of age (Steinbach Hatchery & Feed Ltd., Steinbach, MB, Canada) were used in Studies 1 and 2, respectively. Hens from both studies were housed according to the methods described in our previous work (Goldberg et al. 2012), with approval from the University of Manitoba's Animal Care Protocol Review Committee, in accordance with recommendations established by the Canadian Council on Animal Care (Canadian Council on Animal Care 1993).

### Diets

The isoenergetic and isonitrogenous diets used in Studies 1 and 2 are shown in Tables 1 and 2, respectively. Diets were formulated to meet the requirements of laying hens consuming ∼100 g of feed per day (National Research Council 1994). Diets were predominantly a wheat–soybean meal and barley basal mix, using limestone as the calcium source. Diets from Study 1 were prepared by including 8%, 14%, 20%, and 28% LA (expressed as a percentage of total fatty acids). The last treatment group contained 14% LA with double the amount of SFA. All diets were designed to contain 30% ALA (expressed as a percentage of total fatty acids) in order to produce eggs with an omega-3 nutrient content claim in Canada, which is at least 300 mg omega-3 PUFAs per egg (Health Canada 2012b). The changing LA levels in the diet were achieved by varying the designer oil blends in each diet, which consisted of corn, canola, flaxseed, and high oleic sunflower oils, and the SFA in the last treatment was increased by using lard. Diets from Study 2 were prepared by including two levels of ALA: 15% or 30% (expressed as a percentage of total fatty acids), and three levels of SFA:LA+OA ratio: low (0.5), medium (1.0), or high (2.0). This was achieved by varying the designer oil blends in each diet, which consisted of corn, flaxseed, and nonhydrogenated coconut oil. Due to the high fat content of the diets, an additional 150 international units (IU) alpha-tocopherol (vitamin E) per kg feed was incorporated into all diets as part of the 2.5% vitamin–mineral premix.

**Table 1:** Ingredient composition of layer diets composed of varying levels of linoleic acid (Study 1)

	Dietary treatment				
	
	Linoleic acid (% of total fatty acids)				
	
	8	14	20	28	14
Ingredients (%)					
Ground wheat	31.82	31.82	31.82	31.82	31.82
Ground soybean meal	23.57	23.57	23.57	23.57	23.57
Ground barley	22.15	22.15	22.15	22.15	22.15
Limestone	10.46	10.46	10.46	10.46	10.46
Canola oil	0.01	3.06	3.14	0.97	1.53
Corn oil	0.00	0.00	0.88	2.73	0.19
Flaxseed oil	4.00	3.51	3.48	3.80	3.72
High oleic sunflower oil	3.49	0.94	0.00	0.00	0.00
Lard	0.00	0.00	0.00	0.00	2.06
VM premix^1^	2.50	2.50	2.50	2.50	2.50
Dicalcium phosphate	1.59	1.59	1.59	1.59	1.59
Salt	0.32	0.32	0.32	0.32	0.32
Lysine	0.06	0.06	0.06	0.06	0.06
DL-methionine	0.04	0.04	0.04	0.04	0.04
Calculated composition					
AMEn (poultry; kcal/kg)	2866	2865	2874	2895	2846
Crude protein (%)	18.50	18.50	18.50	18.50	18.50
Crude fat (%)	8.88	8.88	8.88	8.88	8.88
Calcium (%)	4.20	4.20	4.20	4.20	4.20
Total lysine (%)	0.90	0.90	0.90	0.90	0.90
Total phosphorus (%)	0.67	0.67	0.67	0.67	0.67
Available phosphorus (%)	0.45	0.45	0.45	0.45	0.45
Sodium (%)	0.15	0.15	0.15	0.15	0.15
Chloride (%)	0.25	0.25	0.25	0.25	0.25
Methionine (%)	0.30	0.30	0.30	0.30	0.30
Threonine (%)	0.65	0.65	0.65	0.65	0.65
ALA (% of total fatty acids)	28.53	28.70	28.69	28.61	28.49
OA (% of total fatty acids)	49.31	44.88	38.42	28.17	33.19
SFA (% of total fatty acids)	9.56	8.61	8.96	10.43	20.17

VM, vitamin–mineral; AMEn, nitrogen-corrected apparent metabolizable energy; ALA, C18:3n3; OA, C18:1n9; SFA, C16:0 + C16:1 + C18:0.

1 Provided per kg of diet: 11,000 IU vitamin A, 3000 IU vitamin D3, 150 IU vitamin E, 3 mg of vitamin K (as menadione), 0.02 mg cyanocobalamin, 6.5 mg riboflavin, 4 mg folic acid, 10 mg calcium pantothenate, 40.1 mg niacin, 0.2 mg biotin, 2.2 mg thiamine, 4.5 mg pyridoxine, 1000 mg choline, 125 mg ethoxyquin (antioxidant), 66 mg Mn (as manganese dioxide), 70 mg Zn (as zinc oxide), 80 mg Fe (ferrous sulfate), 10 mg Cu (as copper sulfate), 0.3 mg Se (as sodium selenite), 0.4 mg I (as calcium iodate), and 0.67 mg iodized salt.

**Table 2:** Ingredient composition of layer diets composed of two levels of ALA and three levels of saturated fatty acids: linoleic and oleic acid ratio (Study 2)

	Dietary treatment					
	15% ALA^1^			30% ALA^1^		
		
	L_a_	M_b_	H_c_	M_b_	L_a_	H_c_
Ingredients (%)						
Ground wheat	31.82	31.82	31.82	31.82	31.82	31.82
Ground soybean meal	23.57	23.57	23.57	23.57	23.57	23.57
Ground barley	22.15	22.15	22.15	22.15	22.15	22.15
Limestone	10.46	10.46	10.46	10.46	10.46	10.46
Corn oil	3.97	2.61	1.25	2.24	1.11	0.00
Flax oil	2.02	2.05	2.09	4.17	4.20	4.22
Coconut oil	1.51	2.84	4.16	1.09	2.20	3.28
VM premix^2^	2.50	2.50	2.50	2.50	2.50	2.50
Dicalcium phosphate	1.59	1.59	1.59	1.59	1.59	1.59
Salt	0.32	0.32	0.32	0.32	0.32	0.32
Lysine	0.06	0.06	0.06	0.06	0.06	0.06
DL-methionine	0.04	0.04	0.04	0.04	0.04	0.04
Calculated composition						
AMEn (poultry; kcal/kg)	2886	2871	2857	2852	2840	2828
Crude protein (%)	18.50	18.50	18.50	18.50	18.50	18.50
Crude fat (%)	8.88	8.88	8.88	8.88	8.88	8.88
Calcium (%)	4.20	4.20	4.20	4.20	4.20	4.20
Total lysine (%)	0.90	0.90	0.90	0.90	0.90	0.90
Total phosphorus (%)	0.67	0.67	0.67	0.67	0.67	0.67
Available phosphorus (%)	0.45	0.45	0.45	0.45	0.45	0.45
Sodium (%)	0.15	0.15	0.15	0.15	0.15	0.15
Chloride (%)	0.25	0.25	0.25	0.25	0.25	0.25
Methionine (%)	0.30	0.30	0.30	0.30	0.30	0.30
Threonine (%)	0.65	0.65	0.65	0.65	0.65	0.65
LA (% of total fatty acids)	32.11	22.78	13.46	23.31	15.49	7.93
OA (% of total fatty acids)	21.08	17.23	13.40	20.24	17.02	13.90
SFA (% of total fatty acids)	26.80	39.83	52.76	21.66	32.54	43.12

ALA, C18:3n3; L_a_, low ratio of saturated fatty acids: linoleic and oleic acids; M_b_, medium ratio of saturated fatty acids: linoleic and oleic acids; H_c_, high ratio of saturated fatty acids: linoleic and oleic acids; VM, vitamin–mineral; AMEn, nitrogen-corrected apparent metabolizable energy; LA, C18:2n6; OA, C18:1n9; SFA, C16:0 + C16:1 + C18:0.

1 Expressed as a percentage of total fatty acids.

2 Provided per kg of diet: 11,000 IU vitamin A, 3000 IU vitamin D3, 150 IU vitamin E, 3 mg vitamin K (as menadione), 0.02 mg cyanocobalamin, 6.5 mg riboflavin, 4 mg folic acid, 10 mg calcium pantothenate, 40.1 mg niacin, 0.2 mg biotin, 2.2 mg thiamine, 4.5 mg pyridoxine, 1000 mg choline, 125 mg ethoxyquin (antioxidant), 66 mg Mn (as manganese dioxide), 70 mg Zn (as zinc oxide), 80 mg Fe (ferrous sulfate), 10 mg Cu (as copper sulfate), 0.3 mg Se (as sodium selenite), 0.4 mg I (as calcium iodate), and 0.67 mg iodized salt.

### Dietary analysis

Feed samples were analyzed for dry matter, crude protein, and crude fat according to established procedures (AOAC International 1995). The fatty acid composition of the test diets were determined using standard gas chromatographic techniques of the fatty acid methyl esters (AOAC International 1990), using C17:1 methyl ester as an internal standard.

### Experimental protocol

Hens were weighed and caged individually in rollout style cages, and allowed an adaptation period of 7 days before being randomly assigned to receive one of five or six dietary treatments (*n* = 8 per treatment) for Studies 1 and 2, respectively. Both studies were 6 weeks in duration. Body weights of hens were recorded at the start of the trial, and at weekly intervals. Feed consumption was determined for the entire week, and average daily feed intake and feed efficiency were calculated. Egg weights were recorded daily and an average egg production rate was calculated. Prior to sensory analysis, eggs were stored in trays in the dark at 4°C overnight.

### Fatty acid analysis

One egg from each hen at 53 weeks of age and 42 weeks of age was chosen for fatty acid analysis from Studies 1 and 2, respectively (Week 5 for both Studies). Egg yolks were separated from the albumen and stored in plastic bags at −80°C until analysis. Fatty acids were extracted from the egg yolk according to the methods of Folch et al. (1957). The fatty acid composition of the yolks was determined in the same manner as the feed (refer to section Dietary analysis).

### Sample preparation

Sensory analysis was conducted on eggs from Study 2 only. The cooking method from Goldberg et al. (2012) was used. In brief, four randomly chosen eggs from hens at 43 weeks of age (Week 6 of the experiment) of similar weight (56.0 ± 2.0 g) from each treatment were pooled, mixed, and poured into glass jars (Tradition Inc., Montreal, QC, Canada) and covered with an aluminum weighing dish (Fisherbrand Cat No. 08-732; Eagle Thermoplastics, Hodgenville, KY). Jars were placed on a metal rack in a stainless steel cooking pot (10 L) filled with 2 L warm water and cooked for ∼15 ± 1 min. No cooking oil or salt was used. All prepared samples were analyzed within 10 min after preparation was complete. Jars were kept at a constant temperature of 55°C in a heated water bath until panelists evaluated the samples. Advantages of using this cooking method include stable temperature control and retention of volatile compounds produced during the cooking process. Therefore, samples are consistently cooked across treatments and panelists may be able to detect sensory changes more easily.

### Sensory analysis

#### Recruitment

Eight panelists (five females, three males, aged between 18 and 45) who were students and staff of the University of Manitoba were recruited to participate in the sensory evaluation. The sensory analysis component of the research received ethical approval from The University of Manitoba's research ethics board. The criteria for participation included open availability, an interest in the panel, and no aversion or allergies to eggs and any ingredients present in any other products to be used for training purposes (Goldberg et al. 2012). Panelist screening for the criteria listed above was conducted through completion of a questionnaire, where written consent was obtained.

#### Descriptive analysis

The protocol followed for descriptive analysis, which is a modification of the method by Stone and Sidel (2004) can be found in our previous publication (Goldberg et al. 2012). In short, panelists participated in a total of six training sessions in order to sufficiently decrease panelist variability (data not shown) before commencing the test sessions. Egg samples were prepared as described above (see section Sample preparation), and presented coded with a randomly selected three-digit number. During each training session, panelists evaluated the aroma and flavor of each of the randomized samples and developed an agreed vocabulary of attributes, consisting of four aroma and four flavor attributes (Table 3). Cooked commercial eggs and dairy products were also evaluated and used as reference points for each attribute.

**Table 3:** Aroma and flavor definitions and standard products

Attribute	Definition	Standard product^1^/amount
Aroma		
Egg	Aroma associated with whole egg	Blended commercial egg cooked and presented as experimental samples(Canada Safeway, Grade A large, Winnipeg MB)/15 g
Creamy	Aroma associated with whipping cream	Whipping cream (Canada Safeway, Lucerne Brand, Winnipeg, MB)/10 g
Buttery	Aroma associated with unsalted butter	Unsalted butter (Canada Safeway, Lucerne Brand, Winnipeg, MB)/5 g
Sweet	Aroma associated with 2% milk	2% milk (Canada Safeway, Lucerne Brand, Winnipeg, MB)/5 g
Flavor		
Egg	Flavor associated with whole egg	Blended commercial egg cooked and presented as experimental samples (Canada Safeway, Grade A large, Winnipeg MB)/15 g
Creamy	Flavor associated with whipping cream	Whipping cream (Canada Safeway, Lucerne Brand, Winnipeg, MB)/5 g
Buttery	Flavor associated with unsalted butter	Unsalted butter (Canada Safeway, Lucerne Brand, Winnipeg, MB)/5 g
Sweet	Flavor associated with 2% milk	2% milk (Canada Safeway, Lucerne Brand, Winnipeg, MB)/5 g

1 Placed in a 60-mL plastic portion cup and capped with plastic lid about 1 h prior to evaluation and served at room temperature; except for butter samples at 4°C.

On the days of test panels, evaluations were conducted in individual partitioned work stations equipped with SIMS 2000 (2010) computerized sensory software (Sensory Integrated Management System, Morristown, NJ) at the sensory laboratory at the University of Manitoba. Additionally, red lighting was used in work stations to mask any potential color differences between samples. The order of tasting between and within days was balanced to account for first-order and carry-over effects. The sensory attributes of different samples were scored on unstructured 15-cm line scales from 0 (low) to 15 (high). Each panelist was provided with filtered, room temperature water to cleanse their palate between tastings. Panelists were directed to evaluate aroma attributes first, followed by flavor attributes. Egg evaluations were replicated three times on three separate days within the same week, and six samples were assessed at each of the tasting sessions.

### Statistical analysis

Both studies were designed to be completely randomized, and all analyses were conducted using SPSS Statistics version 20.0 (SPSS Inc., Chicago, IL). Fatty acids were analyzed using one-way and two-way analysis of variance (ANOVA) for Studies 1 and 2, respectively. Sensory data were analyzed using three-way ANOVA. The model included Dietary Treatments (T) and Panelists (P) as fixed effects and Replications (R) as a random effect, and two-way interactions of Panelist by Dietary Treatment, Dietary Treatment by Replication, and Panelist by Replication. When interactions were not significant they were pooled with the error (O′Mahony 1986). *F*-values were recalculated with the additional sums of squares for error and the corresponding degrees of freedom. If a significant panelist-by-treatment interaction was observed, the main effects were tested by the interaction effect (Stone et al. 2012). Fisher's least significant difference test was used to determine mean treatment differences when significant (*P* < 0.05). Partial least squares (PLS) (XLSTAT version 2012; Addinsoft, Paris, France) analysis was used to generate a biplot by using average values for all attributes of interest to provide a visual perspective of the correlation between the samples in relation to the corresponding yolk fatty acids and sensory attribute intensities.

## Results and Discussion

### Bird health

Mean values across treatments including the control from each of Studies 1 and 2 were not significantly different (*P* ≥ 0.05) from one another. The following data are expressed as mean values ± the standard error of the mean (SEM) for Studies 1 and 2, respectively. Egg production per week was 6.2 ± 0.10 and 6.6 ± 0.09, egg weight was 62.6 g ± 0.61 and 58.4 g ± 0.45, average feed consumption was 90.8 ± 1.58 and 97.0 ± 1.56 g/day, and feed conversion efficiency was 1.45 g of feed/g of egg ±0.02 and 1.67 g of feed/g of egg ±0.03. Birds from Study 1 initially weighed 1.8 ± 0.01 kg, and by the end of the trial they weighed 1.7 ± 0.04 kg. Birds from Study 2 initially weighed 1.7 ± 0.01 kg, and by the end of the trial they weighed 1.6 ± 0.02 kg.

### Fatty acid composition of egg yolks

Yolk fatty acid analysis from Studies 1 and 2 are shown in Tables 4, 5, respectively. In Study 1, omega-6 PUFAs, oleic acid (OA), EPA, and DPA were affected by dietary treatment. An increasing level of dietary LA led to a decrease in yolk OA while LA, gamma-linolenic acid (GLA), arachidonic acid (AA), and total omega-6 PUFAs were increased. This observation was expected because LA in the diet is increased at the expense of OA. Increasing LA in the diet also led to a decrease in EPA with an increase in DPA. Doubling the SFA content of the 14% LA treatment did not affect the yolk fatty acid content. The increase in DPA was rather unexpected, as our hypothesis was that suppression of dietary LA would lead to a greater amount of ALA conversion due to preferential desaturation of ALA. Furthermore, we also expected to see a decrease in yolk DHA as LA increased; however, the differences between treatments in dietary LA were not large enough to change DHA levels. Altering the ratio of omega-6:omega-3 PUFA or the absolute amount of ALA might have a more profound effect, as we have found in a previous work (Goldberg et al. 2012), mainly due to the larger differences in ALA content. Study 2 attempted to answer this question while proposing an alternative method to alter the dietary fatty acid composition.

**Table 4:** Fatty acid composition of egg yolks from hens fed varying levels of linoleic acid (Study 1)

Fatty acid (mg/g yolk)	Dietary treatment					SEM	*P*-value

	Linoleic acid (% of total fatty acids)						

	8	14	20	28	14^1^		
PALM	45.1	44.2	44.7	44.9	43.9	0.65	NS
PALMO	6.4	6.0	5.7	5.4	5.5	0.19	NS
SA	15.3	17.0	16.4	18.7	16.8	0.37	NS
OA	99.0^b^	93.2^ab^	85.3^ab^	83.2^ab^	79.6^a^	1.61	*
LA	24.4^a^	28.8^ab^	35.1^bc^	36.3^c^	29.8^abc^	0.98	***
GLA	0.2^a^	0.3^ab^	0.3^ab^	0.4^b^	0.3^ab^	0.01	**
AA	2.3^a^	2.5^ab^	2.8^ab^	3.0^b^	2.5^ab^	0.08	*
ALA	9.5	9.9	10.2	9.2	10.0	0.31	NS
EPA	0.3^b^	0.3^b^	0.3^b^	0.2^a^	0.3^b^	0.01	***
DPA	0.4^a^	0.4^a^	0.5^ab^	0.6^b^	0.5^ab^	0.02	*
DHA	4.2	3.7	3.9	4.4	3.9	0.07	NS
Total n-6	27.0^a^	31.5^ab^	38.3^bc^	39.6^c^	32.5^abc^	1.05	***
Total n-3	14.4	14.4	14.8	14.4	14.7	0.34	NS

Data within a row with different superscripts are significantly different. PALM, C16:0; PALMO, C16:1; SA, C18:0; OA, C18:1n9; LA, C18:2n6; GLA, C18:3n6; AA, C20:4n6; ALA, C18:3n3; EPA, C20:5n3; DPA, C22:5n3; DHA, C22:6n3; Total n-6, C18:2n6 + C18:3n6 + C20:4n6; Total n*-*3, C18:3n3 + C20:5n3 + C22:5n3 + C22:6n3.

1 Diet containing 20% saturated fat (% of total fatty acids).

Levels of significance: NS *P* ≥ 0.05; *P < 0.05, **P < 0.01, ***P < 0.001.

**Table 5:** Fatty acid composition of egg yolks procured from hens fed two levels of ALA and three levels of saturated fatty acids: linoleic and oleic acid ratio (Study 2)

	Dietary treatment									
			
	15% ALA^1^			30% ALA^1^				*P*-value		
				
Fatty acid (mg/g yolk)	L_a_	M_b_	H_c_	L_a_	M_b_	H_c_	SEM	Ratio	ALA	Ratio × ALA
LAUR	0.2	0.6	0.8	0.2	0.3	0.6	0.04	***	***	NS
MYRIST	2.6	4.8	6.2	0.9	3.3	5.7	0.26	***	**	NS
PALM	46.4^b^	48.3^b^	46.4^b^	41.0^a^	43.3^a^	48.1^b^	0.63	*	**	*
PALMO	3.6^b^	4.5^c^	4.5^c^	3.5^ab^	4.0^bc^	6.2^d^	0.18	***	NS	***
SA	17.5	16.2	16.0	17.0	13.9	15.5	0.41	NS	NS	NS
OA	67.9	65.4	63.8	65.6	58.2	65.3	1.26	NS	NS	NS
LA	47.2	43.3	37.7	38.8	35.4	30.0	0.93	***	***	NS
GLA	0.3	0.3	0.3	0.2	0.2	0.2	0.01	NS	***	NS
AA	3.1	2.8	2.6	2.1	2.0	1.6	0.09	***	***	NS
ALA	8.0^a^	8.7^a^	8.6^a^	14.2^b^	15.0^b^	17.9^c^	0.61	**	***	**
EPA	0.2^a^	0.2^a^	0.2^a^	0.3^b^	0.4^c^	0.5^d^	0.02	***	***	**
DPA	0.4	0.4	0.6	0.5	0.6	0.6	0.02	**	*	NS
DHA	2.9	3.1	3.5	2.7	3.1	3.2	0.07	**	NS	NS
TOTAL n-6	50.5	46.4	40.6	41.2	37.6	31.8	1.01	***	***	NS
TOTAL n-3	11.3	12.4	12.9	17.6	19.0	22.2	0.64	***	***	NS

Data within a row with different superscripts are significantly different. L_a_, low ratio of saturated fatty acids: linoleic and oleic acids; M_b_, medium ratio of saturated fatty acids: linoleic and oleic acids; H_c_, high ratio of saturated fatty acids: linoleic and oleic acids; LAUR, C12:0; MYRIST, C14:0; PALM, C16:0; PALMO, C16:1; SA, C18:0; OA, C18:1n9; LA, C18:2n6; GLA, C18:3n6; AA, C20:4n6; ALA, C18:3n3; EPA, C20:5n3; DPA, C22:5n3; DHA, C22:6n3; Total n-6, C18:2n6 + C18:3n6 + C20:4n6; Total n*-*3, C18:3n3 + C20:5n3 + C22:5n3 + C22:6n3.

1 Expressed as a percentage of total fatty acids.

Levels of significance: NS *P* ≥ 0.05; *P < 0.05, **P < 0.01, ***P < 0.001.

Saturated fatty acids were the main focus of diets in Study 2. As such, we incorporated higher levels of SFA into the feed rather than manipulating the omega-6 and -9 fatty acid content. We used coconut oil in Study 2 to serve this function because it is a vegetable source of SFA compared to the use of lard in Study 1. For years, concern over the possible detrimental health effects of coconut oil revolved around its high SFA content (∼90%, expressed as a percentage of total fatty acids) and therefore its subsequent atherogenic effect (Marina et al. 2009). However, many studies have demonstrated improved lipid profiles, with lowered total cholesterol, lipoproteins, and phospholipids with coconut oil supplementation (Nevin and Rajamohan 2004). The reason being that coconut oil is largely composed of medium-chain triacylglycerols (MCTs) (∼65% of total fatty acids in coconut oil), which contain SFA with carbon chain lengths of 6–12 atoms (Marten et al. 2006). MCTs are rapidly absorbed and metabolized in the liver into energy, and unlike long-chain fatty acids they do not participate in cholesterol biosynthesis and transport (Dayrit 2003; Marina et al. 2009). However, lipid metabolism is quite different in laying hens compared to humans. Hens have a poorly developed lymphatic system; therefore, lipoproteins are secreted directly into the portal system (Hermier 1997). Laying hens are able to synthesize their own triglycerides, cholesterol, and phospholipids through de novo hepatic lipogenesis (Hermier 1997).

One of the potential concerns with supplementing the layer diet with high amounts of SFA was the accumulation of even higher amounts in the egg yolk as compared to conventional eggs. We not only observed a significant increase in yolk SFA but also an interaction between ALA and SFA:LA+OA ratio. Therefore, the benefits of improved fatty acid profile in terms of increasing omega-3 PUFA conversion must be weighed against the potential downsides of increased SFA deposition, albeit the increases observed were modest and may not result in a major biological change. Additionally, increasing the SFA:LA+OA ratio led to a substantial decrease in omega-6 PUFAs, and modest increases in the lauric acid (LAUR) content (due to the coconut oil). Human trials are necessary to determine the potential effects of these eggs on indices of health. However, many studies have shown that there are substantial differences among SFA in terms of their cholesterol-raising effects. Investigators are in agreement that the total high-density lipoprotein (HDL) ratio is more sensitive and specific than is total cholesterol as an indicator of atherogenic risk (Stampfer et al. 1991; Kinosian et al. 1995; Assmann et al. 1996; Mensink et al. 2003). A meta-analysis by Mensink et al. (2003) found that despite LAUR being the most potent total and low-density lipoprotein cholesterol-raising SFA, it had the most favorable effect on the total:HDL ratio in human subjects than any other fatty acid, either saturated or unsaturated. After LAUR, the total:HDL ratio was more favorably affected by stearic acid (SA), myristic acid (MYRIST), and palmitic acid (PALM), in order from most to least favorable.

Our findings from Study 2 are in agreement with Wignjosoesastro et al. (1972), where they found an addition of 10% coconut oil in the layer diet led to increased LAUR and MYRIST and decreased SA, OA, and LA in the yolk fat fraction. The only difference was that our diets were not able to decrease SA and OA due to the significantly lower level of coconut oil inclusion (the highest level of inclusion in our diets was 4.16% in the H1 group) in combination with flax and corn oils. In fact, dietary treatments had no effect on the content of SA and OA in the egg yolk.

Although LAUR is an MCT found in large quantities in coconut oil (∼48%), only minor amounts were detected in the yolk. Whereas, MYRIST (a common SFA found in ∼19% in coconut oil) resulted in much larger quantities in the yolk compared to LAUR. It is evident that the rapid utilization of MCTs for energy resulted in minor deposition into yolk. Human and animal studies have shown that MCTs are preferentially oxidized, in turn, leading to increased thermogenesis (Baba et al. 1982; Hill et al. 1989; Kasai et al. 2002; Noguchi et al. 2002). Furthermore, isotopic tracer and serum fatty acid analyses (Nagata et al. 2003; Rodriguez et al. 2003) suggest that MCTs might be able to spare oxidation of PUFAs. This hypothesis could offer an explanation of the greater levels of yolk omega-3 PUFAs in Study 2 eggs compared to Study 1 eggs, despite the same level of ALA in the diet (30% ALA). Further investigation using hens of the same age and strain is needed to fully elucidate this finding.

Excluding SA and OA, the level of ALA in the diet affected all other fatty acids, with the exception of palmitoleic acid (PALMO) and DHA, and the SFA:LA+OA ratio affected all other fatty acids except GLA. Increasing dietary ALA resulted in marked decreases in the remaining fatty acids; however, significant increases were found in ALA, EPA, DPA, and total omega-3 PUFAs. Total omega-3 PUFA content was highest in the high ratio groups, with the higher ALA group containing the most (22.2 mg total omega-3 PUFA/g of yolk); however, better LCPUFA deposition was found in the lower ALA group, which contained 3.5 mg DHA/g of yolk. Increasing the dietary ALA content above 15% did not affect yolk DHA. In fact, slight decreases were observed in the 30% ALA group. This suggests that retro conversion of DHA back to DPA and EPA may have occurred, considering increases in these fatty acids were observed in the 30% group, or that ALA conversion was suppressed, or both. Conversely, increasing the SFA:LA+OA ratio resulted in marked increases in the remaining fatty acids; however, significant decreases were found in LA, AA, and total omega-6 PUFAs. In this case, DHA did increase with an increasing SFA:LA+OA ratio, suggesting that the LA:ALA ratio should not be the only consideration when attempting to enhance ALA conversion to LCPUFAs. A significant interaction (*P* < 0.05) between the ratio and ALA was observed in PALM, PALMO, ALA, and EPA, with total omega-3 PUFAs close to significance at *P* = 0.056.

The omega-6:omega-3 PUFA ratio in the eggs from diets in Study 2 decreased with (1) increasing dietary SFA:LA+OA ratio and (2) increasing dietary ALA. According to Simopoulos (2002), humans evolved on a diet in which the ratio of omega-6:omega-3 PUFA was about 1, whereas the ratio in Western diets is 15–17. Conventional eggs typically have an omega-6:omega-3 PUFA ratio even higher than the typical Western diet, at ∼20. All of the eggs in this study were within the suggested optimal range of 1–4 for the ratio of omega-6:omega-3 PUFA, with the H2 group having the lowest ratio at 1.4.

### Sensory analysis

Results from descriptive analysis on cooked eggs from Study 2 are shown in Table 6. A highly significant difference (*P* < 0.001) was observed over the range of scores by individual panelists. Although training helps to decrease the panelist variability due to different sensitivities and use of the line scale, it cannot completely eliminate it (Lundahl and McDaniel 1988). Interaction effects were observed between panelist and replication for creamy and sweet aromas, and creamy, buttery, and sweet flavors. This interaction was investigated and not one particular panelist was responsible for the inconsistency. Creamy flavor was the only attribute that showed a significant panelist × treatment interaction. In all samples, the most intense aroma and flavor attribute was described as “egg,” which is in accord with our previous findings (Goldberg et al. 2012).

**Table 6:** Descriptive analysis results from three-way ANOVA (T = Dietary treatment [*n* = 6]; P = Panelist [*n* = 8]; R = Replication [*n* = 3]) for cooked eggs procured from hens fed two levels of ALA and three levels of saturated fatty acids: linoleic and oleic acid ratio (Study 2)

				Dietary treatment					
		
				Mean intensity ratings – 0- to 15-cm line scale (SEM)					
		
	Source of variation (*F*-value)			15% ALA^1^			30% ALA^1^		
			
	T	P	R	L_a_	M_b_	H_c_	L_a_	M_b_	H_c_
Aroma									
Egg	0.79 NS	43.26***	2.65 NS	8.1 (0.8)	9.0 (0.8)	9.1 (0.6)	9.0 (0.6)	9.0 (0.8)	8.9 (0.9)
Creamy	1.01 NS	14.15***	0.77 NS	3.5 (0.5)	3.4 (0.6)	3.9 (0.6)	3.9 (0.5)	3.7 (0.6)	4.3 (0.8)
Buttery	0.19 NS	50.53***	0.49 NS	4.8 (0.7)	5.0 (0.7)	5.2 (0.7)	5.3 (0.8)	5.3 (0.7)	5.3 (0.9)
Sweet	0.82 NS	9.03***	1.58 NS	1.6 (0.3)	2.4 (0.6)	2.1 (0.6)	1.9 (0.5)	1.9 (0.5)	2.1 (0.6)
Flavor									
Egg	1.50 NS	21.74***	1.07 NS	9.3 (0.7)	8.4 (0.8)	9.8 (0.6)	9.1 (0.7)	10.1 (0.7)	8.9 (0.8)
Creamy	1.35^2^ NS	11.95^2^ ***	0.08 NS	4.7 (0.7)	3.1 (0.6)	3.4 (0.4)	3.4 (0.6)	3.4 (0.5)	4.0 (0.6)
Buttery	1.33 NS	13.38***	0.21 NS	5.6 (0.8)	4.3 (0.7)	4.5 (0.5)	4.4 (0.7)	4.4 (0.6)	5.1 (0.8)
Sweet	1.07 NS	34.14***	0.64 NS	1.6 (0.3)	1.9 (0.4)	1.7 (0.4)	1.8 (0.4)	1.4 (0.3)	1.6 (0.3)

Interaction *F*-values and significance not shown in Table; PxT, Creamy flavor 1.65*; PxR, Creamy aroma 2.91***; Sweet aroma 5.65***; Creamy flavor 1.82*; Buttery flavor 1.88*; Sweet flavor 2.32**. ANOVA, analysis of variance; ALA, alpha-linolenic acid; L_a_, low ratio of saturated fatty acids: linoleic and oleic acids; M_b_, medium ratio of saturated fatty acids: linoleic and oleic acids; H_c_, high ratio of saturated fatty acids: linoleic and oleic acids.

1 Expressed as a percentage of total fatty acids.

2 New *F*-value as determined by testing the main effects by the interaction effect.

Levels of significance: NS *P* ≥ 0.05; *P < 0.05, **P < 0.01, ***P < 0.001.

No significant differences in sensory attributes between dietary treatments for cooked egg samples were found. It is possible that the reason for this is that the level of vitamin E included in these diets was adequate to prevent oxidation that would result in aroma and flavor changes. Limited data are available on the sensory effects of eggs from hens supplied with high doses of vitamin E in their diet. However, Leeson et al. (1998) found that 100 IU compared to 10 IU vitamin E/kg of diet accentuated the effects of decreased acceptability in boiled eggs from hens fed 20% flaxseed, but not in hens fed a control diet without flaxseed. The authors suggested that the higher dose of vitamin E may have resulted in a pro-oxidant effect that led to decreased acceptability. In contrast, Franchini et al. (2002) found that an even greater dose of 200 IU vitamin E/kg of diet did not alter sensory perception compared to hens fed no additional vitamin E. However, both studies utilized an untrained consumer panel, whereas a trained descriptive panel was used to assess specific sensory attributes in this study. Also, differences in the cooking and sensory testing methods could have resulted in such differences to be significant. The degree of vitamin E supplementation in the current study was unchanged across dietary treatments. Because significant sensory differences in the resultant eggs were not observed, the possibility of this level of vitamin E causing sensory changes, particularly negative, is not reasonable. Our results in this regard align with our objective of observing nonsignificant aroma and flavor changes. Although improving the sensory profile of enriched eggs would be ideal, for instance, as an increase in egg flavor, this would be difficult to achieve.

**Figure 1:**
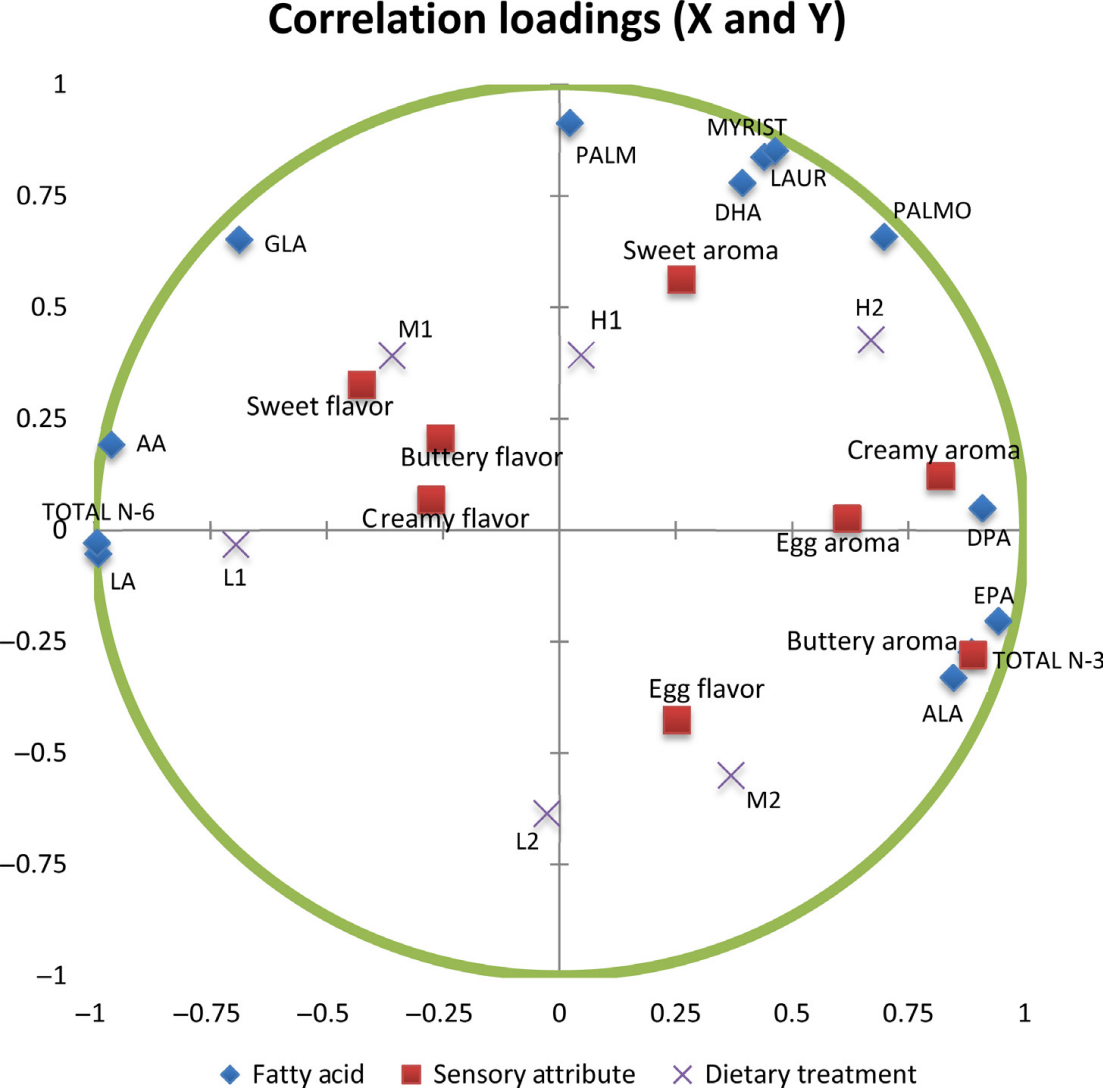
Correlation loadings from partial least squares (PLS) analyses between yolk fatty acids (*X*-variables) and sensory attributes of the cooked egg samples (*Y*-variables). L, low; M, medium; H, high ratio of saturated fatty acids: linoleic and oleic acids; 1, 15% ALA; 2, 30% ALA (expressed as a percentage of total fatty acids); LAUR, C12:0; MYRIST, C14:0; PALMO, C16:1; LA, C18:2n6; GLA, C18:3n6; AA, C20:4n6; ALA, C18:3n3; EPA, C20:5n3; DPA, C22:5n3; DHA, C22:6n3; TOTAL n-6, C18:2n6 + C18:3n6 + C20:4n6; TOTAL n-3, C18:3n3 + C20:5n3 + C22:5n3 + C22:6n3.

In Figure 1, the graphical presentation of the correlation loadings using PLS depicts overall possible relationships between yolk fatty acids and sensory attributes of cooked eggs. The first observation to note is the clear separation between dietary treatments. Based on the greater separation between the ALA groups (*n* = 2) compared to the SFA:LA+OA ratio groups (*n* = 3), it seems that dietary ALA plays a larger role in the sensory outcomes. There also appears to be similar separations between the 2 L and 2 H treatments. However, a much larger separation exists between the M treatments, suggesting a greater sensory difference between them. In terms of fatty acids, there are three main clusters. The first cluster found closer to the low ALA groups consists of all omega-6 PUFAs, including LA, AA, and GLA. The second, which is closer to the high ratio groups, is DHA, LAUR, MYRIST, and PALMO. The third, which is found closer to the high ratio groups, are the omega-3 PUFAs, including ALA, EPA, and DPA. The most interesting finding is that DHA is more related to SFA as opposed to the other three omega-3 PUFAs. With regard to the association between fatty acids and sensory attributes, there are four main observations. First, egg flavor appears to be closely related to the omega-3 PUFAs, a finding that was corroborated in our previous study analyzing the sensory differences from eggs procured from hens fed hemp (Goldberg et al. 2012). The difference in this study is that DHA was unrelated to the other omega-3 PUFAs, which was not the case in Goldberg et al. (2012). Second, egg, creamy, and buttery aromas are more related to omega-3 PUFAs, and third, sweet, buttery, and creamy flavors are more related to the omega-6 PUFAs. Lastly, sweet aroma is more related to SFA and DHA. Based on these observations, there is a possibility that the omega-3 PUFAs and SFA are more closely related to aromas, whereas omega-6 PUFAs to flavors.

## Conclusions

The present research objectives were to assess the impact of altering the LA content and the SFA:LA+OA ratio in laying hen diets on the conversion of omega-3 PUFAs into the resultant eggs. For the latter study, there was an additional objective of examining the potential for changes in the sensory attributes of the cooked eggs due to a greater change in yolk fatty acids. These objectives were addressed by testing egg yolks for fatty acid content and by utilizing a trained sensory panel to determine the aroma and flavor attribute intensities of cooked eggs.

The amount of ALA and LA in the diet and the dietary ratio of SFA:LA+OA play a major role in the fatty acid composition of resultant eggs. The most important observations to note from the current research are (1) that increasing the dietary LA content led to a decrease in EPA and an increase in DPA and (2) that the SFA:LA+OA ratio plays more of a role in ALA conversion into LCPUFAs, especially with regard to DHA, compared to increasing ALA content alone. Despite differences in the fatty acid composition of the eggs, there were no sensory differences in cooked eggs across all dietary treatments. However, egg flavor appeared to be positively correlated with omega-3 PUFAs, with the exception of DHA. This suggests that there may be potential to alter the sensory profile in cooked eggs by altering the SFA:LA+OA ratio in the diet. It also suggests that the addition of preformed DHA in the laying hen diet may not impact egg flavor, but this does not rule out the possibility that other aromas or flavors may be developed. Further research is necessary to understand the close association between egg flavor and omega-3 PUFAs, and before this can be achieved, it is essential that egg flavor be clearly defined in terms of the mixture of volatiles that compose it.

In summary, the differences in LA in Study 1 diets were not large enough to result in any major impact on ALA conversion. Results from Study 2 show that it is possible to incorporate modest amounts of LAUR into egg yolk through coconut oil feeding and that a higher SFA:LA+OA ratio modestly increases the conversion of ALA into LCPUFAs without negatively affecting the sensory quality in cooked table eggs. Based on this research, a high SFA:LA+OA ratio (using coconut oil as the SFA source) coupled with a low omega-6:omega-3 PUFA ratio in laying hen diets is recommended to enhance ALA conversion, MCT deposition, and decrease omega-6 PUFAs in the egg yolk.
